# Supplementary material for: Inflammatory factors and risk of lung adenocarcinoma: a Mendelian randomization study mediated by blood metabolites
Source: Front Endocrinol (Lausanne). 2024 Aug 27;15:1446863. doi: 10.3389/fendo.2024.1446863 (PMC11384989; doi:10.3389/fendo.2024.1446863)
Supplement: Supplementary file 1 [file DataSheet1.zip › supplementary files/S2/GCST90200205/forest.pdf]

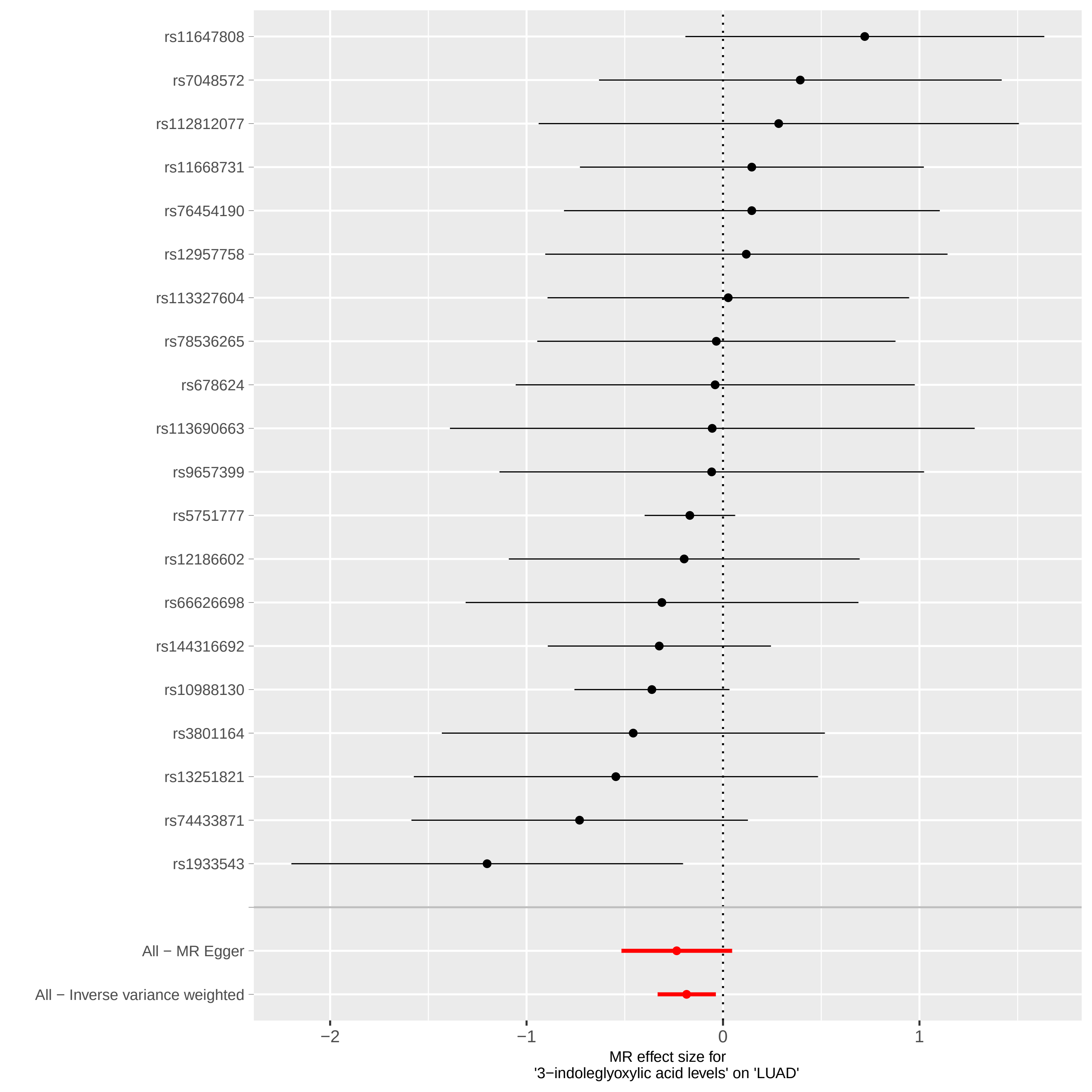
rs11647808
rs7048572
rs112812077
rs11668731
rs76454190
rs12957758
rs113327604
rs78536265
rs678624
rs113690663
rs9657399
rs5751777
rs12186602
rs66626698
rs144316692
rs10988130
rs3801164
rs13251821
rs74433871
rs1933543
All − MR Egger
All − Inverse variance weighted
−2
−1
0
1
MR effect size for
'3−indoleglyoxylic acid levels' on 'LUAD'
